# Routine Fundoscopy Uncovering Wolfram Syndrome in a Diabetic Patient: A Case Report

**DOI:** 10.7759/cureus.101313

**Published:** 2026-01-11

**Authors:** Hatim Bazhar, Moulahid Loubna, Nabil Bouslous, Moustaine Omar

**Affiliations:** 1 Ophthalmology, Souss Massa University Hospital, Agadir, MAR

**Keywords:** diabetes insipidus, diabetes mellitus, optic atrophy, sensorineural deafness, wolfram syndrome

## Abstract

Wolfram syndrome is a rare inherited neurodegenerative disorder, in which early ophthalmologic abnormalities may provide the initial diagnostic clue. In this article, we report the case of a 20-year-old male with early‐onset bilateral deafness and insulin-dependent diabetes mellitus who was referred for evaluation of possible diabetic retinopathy. He reported no visual complaints at the time of presentation. Examination showed a visual acuity of 2/10 bilaterally, and optic disc pallor on fundoscopy without diabetic retinopathy. Further complementary testing revealed diffuse visual field defects and marked retinal nerve fiber layer (RNFL) thinning on optical coherence tomography (OCT), consistent with optic atrophy. Neuroimaging showed posterior pituitary agenesis, and subsequent endocrine evaluation confirmed diabetes insipidus. Taken together with the patient’s sensorineural deafness and diabetes mellitus, these findings strongly supported the diagnosis of Wolfram syndrome. Genetic testing was advised but not performed due to financial limitations. Ophthalmic management consisted of vitamin supplementation, low-vision rehabilitation, and scheduled follow-up. Given the multisystemic nature of the disease, multidisciplinary follow-up was implemented. This case emphasizes the importance of broad systemic workup in young individuals with atypical ocular findings and highlights Wolfram syndrome as a key differential diagnosis when optic atrophy is accompanied by hearing loss or diabetes mellitus.

## Introduction

Wolfram syndrome, also known as DIDMOAD (diabetes insipidus, diabetes mellitus, optic atrophy, and deafness), is a rare and progressive neurodegenerative condition most commonly associated with pathogenic variants in the *WFS1 *gene [1]. It typically presents in childhood, with early-onset diabetes mellitus and optic atrophy as hallmark initial manifestations [1,2]. Despite its rarity, this disease is marked by considerable morbidity and mortality owing to the lack of treatments that can halt or reverse its progression [1,2]. This report presents a case of a young patient whose clinical findings suggested a diagnosis of Wolfram syndrome following a comprehensive ophthalmologic and systemic evaluation.

## Case presentation

A 20-year-old North African male, the only child of non-consanguineous parents, with insulin-dependent diabetes mellitus, was referred for an ophthalmologic evaluation to screen for diabetic retinopathy. His medical history included untreated bilateral sensorineural hearing loss since early childhood. He reported no visual complaints at presentation.

**Figure 1:**
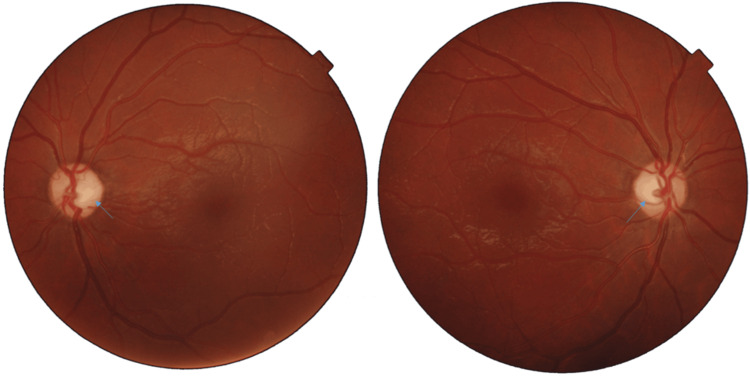
Bilateral pallor of the optic disc. The images show more pronounced temporally (blue arrows), without any signs of diabetic retinopathy in either eye.

On examination, best-corrected visual acuity (Snellen equivalent) measured 2/10 in the right eye (Ocular dexter (OD)) and 2/10 in the left eye (Ocular sinister (OS)), with symmetric and reactive pupils. Ocular motility, adnexal structures, and anterior segment assessment were unremarkable. Intraocular pressure was 13 mmHg in OD and 12 mmHg in OS. Fundus examination revealed bilateral optic disc pallor with no evidence of diabetic retinopathy (Figure 1).

**Figure 2:**
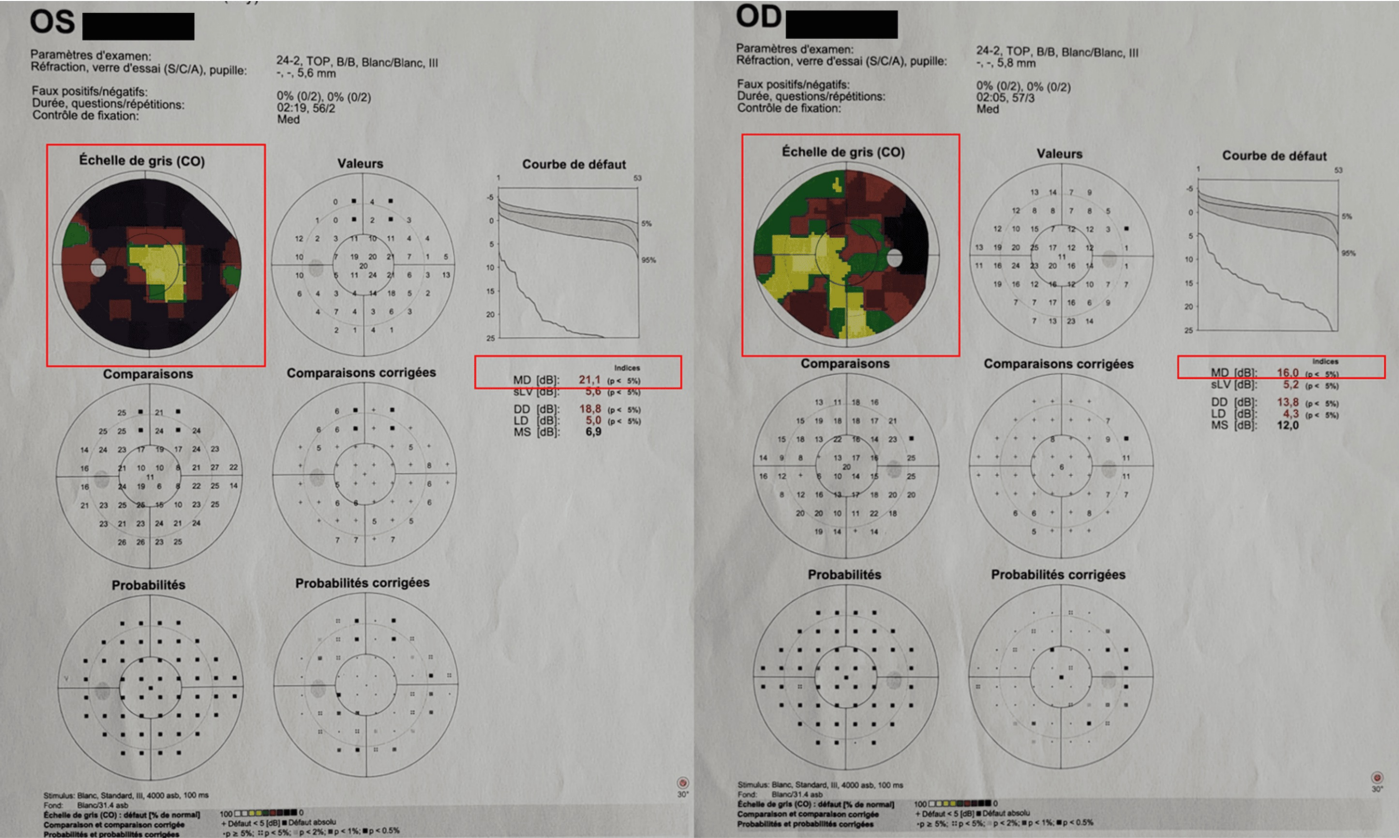
Octopus visual field testing. The images show markedly advanced and deep defects in both eyes (red rectangles), predominating in the left eye (mean deviation: 21.1 dB).

In view of these findings, complementary investigations were performed. Visual field testing (Octopus) showed severe and diffuse central and peripheral defects in both eyes (Figure 2).

**Figure 3:**
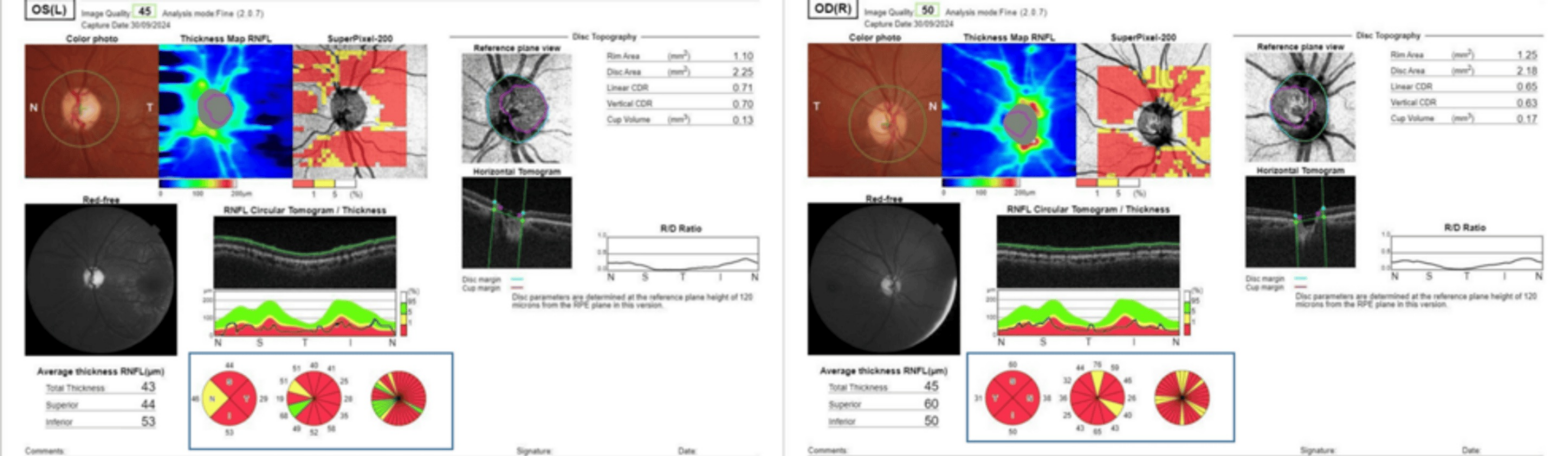
RNFL mapping. The images show significant bilateral thinning affecting all four quadrants (blue rectangle), with the temporal quadrant being most severely affected(38 µm in the right eye and 29 µm in the left). RFNL: retinal nerve fiber layer.

Optical coherence tomography (OCT) demonstrated marked RNFL thinning in all four quadrants bilaterally (Figure 3).

**Figure 4:**
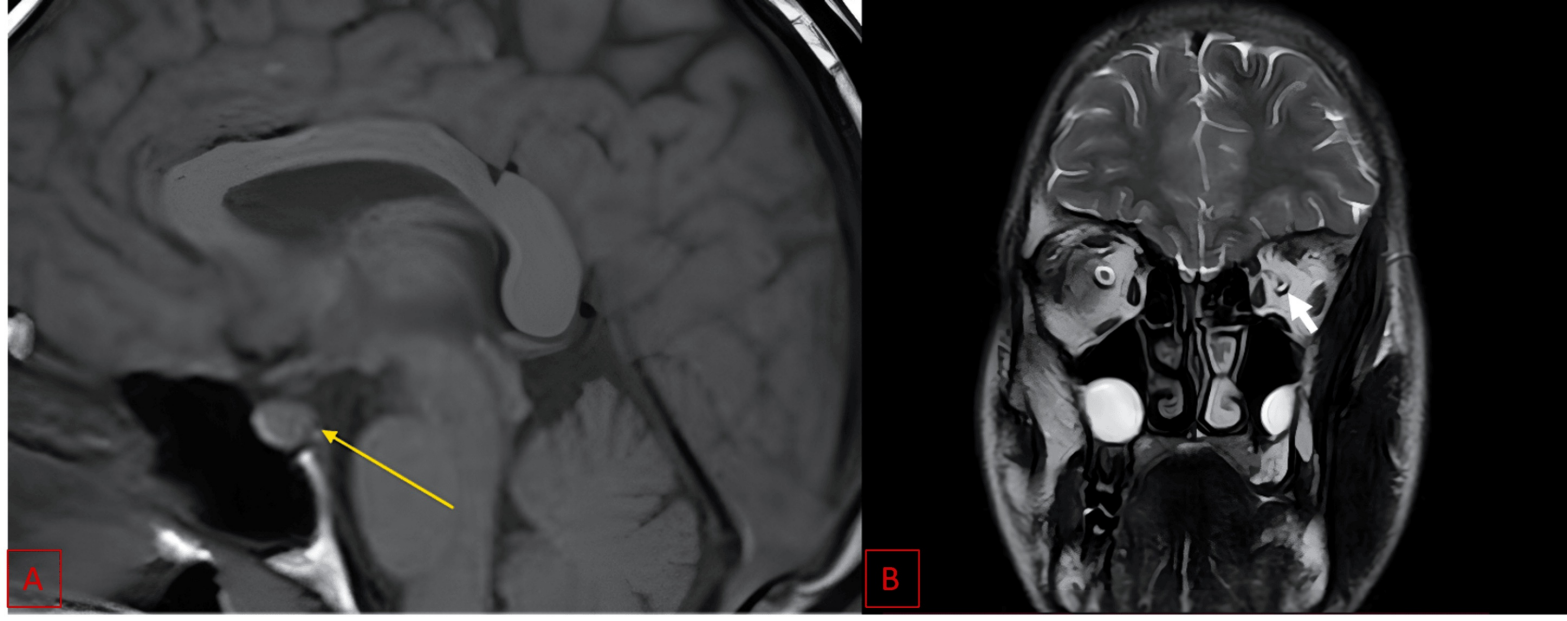
Saggital and Coronal MRI images. A: Sagittal T1-weighted brain MRI shows absence of the normal hyperintense posterior pituitary bright spot (yellow arrow), consistent with posterior pituitary agenesis. B: Coronal T2-weighted images demonstrate bilateral optic nerve atrophy, more marked on the left (white arrow), with no evidence of compressive or infiltrative lesions.

 Brain MRI excluded compressive or infiltrative lesions but revealed posterior pituitary agenesis (Figure 4).

Given the abnormal pituitary findings on imaging, an endocrinologic evaluation was requested with subsequent laboratory tests (urine osmolality and desmopressin response) that confirmed associated diabetes insipidus. In parallel, otorhinolaryngologic assessment verified the central origin of the patient’s deafness.

The combination of optic atrophy, central sensorineural deafness, diabetes mellitus and diabetes insipidus strongly supported the diagnosis of Wolfram syndrome type 1. Our management included vitamin supplementation, low-vision therapy, and long-term ophthalmic follow-up. A multidisciplinary plan was implemented, incorporating auditory rehabilitation and ongoing endocrine care. Genetic testing was recommended to identify the causal mutation, but financial limitations prevented its completion.

## Discussion

Wolfram syndrome is a rare, progressive neurodegenerative disorder, typically inherited in an autosomal recessive manner [2]. It is typically associated with mutations in the *WFS1* gene, although other mutations, such as *WFS2,* can lead to similar presentations [1-3]. Its key features include diabetes mellitus, optic atrophy, and hearing loss, with potential involvement of diabetes insipidus (especially in type 1), urological, neurological, and psychiatric abnormalities [1,4,5].

Optic atrophy, a hallmark of Wolfram Syndrome, is seen in nearly all cases and is critical for diagnosis. Other ocular manifestations may include cataracts, strabismus, glaucoma, and pigmentary retinopathy [5,6]. Notably, diabetic retinopathy is rare, possibly due to retinal vessel attenuation from optic atrophy, which may protect the retina from glucose toxicity [6]. While no cure exists, ongoing research into regenerative treatments, gene therapy, and Idebenone for optic atrophy shows promise [1,5,6]. The clinical course of Wolfram syndrome is highly variable and often unpredictable. In this patient, close and regular monitoring was implemented given the absence of curative treatment options [7,8,9]. Early diagnosis may facilitate timely multidisciplinary care, although the prognosis remains poor due to disease progression and severe neurological impairments [1,2,7,10].

This case report has several limitations. Genetic confirmation of Wolfram syndrome was not performed due to financial constraints, and the diagnosis was therefore based on clinical, ophthalmological, neuroimaging, and endocrine findings. Moreover, the absence of long-term ophthalmologic and systemic follow-up at the time of reporting limits assessment of disease progression.

## Conclusions

This case illustrates how ophthalmic evaluation revealed an underlying systemic disorder, contributing to earlier diagnostic suspicion and clinical assessment. In this patient, the presence of symmetric optic neuropathy prompted consideration of Wolfram syndrome after more common etiologies had been excluded. A comprehensive evaluation, including audiometric testing, endocrine assessment, and genetic counselling, was performed to better characterize the condition. This case also underscores the potential value of multidisciplinary follow-up in addressing the progressive nature of the disease.
